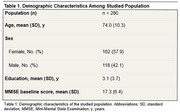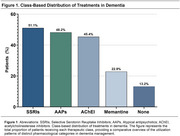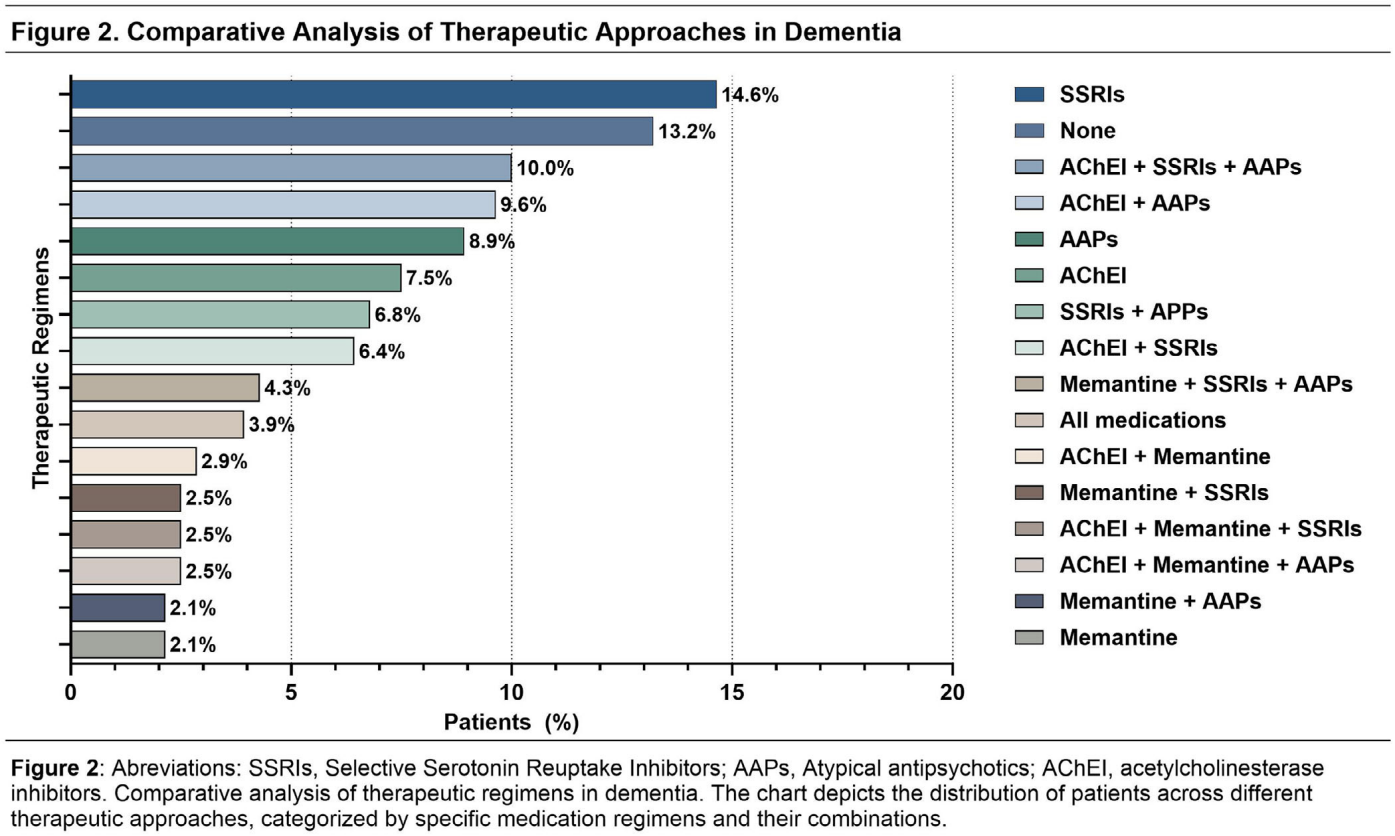# Prescription Trends in Dementia Care: A 10‐Year Review from a Brazilian Public Memory Clinic Perspective

**DOI:** 10.1002/alz70860_106549

**Published:** 2025-12-23

**Authors:** Carlos Gustavo Heyer Lokschin, Fernando Jacob Lazzaretti, Cristiano Aguzzoli, Lucas Porcello Schilling

**Affiliations:** ^1^ School of Medicine, Pontifícia Universidade Católica do Rio Grande do Sul (PUCRS), Porto Alegre, Rio Grande do Sul, Brazil; ^2^ Brain Institute of Rio Grande do Sul (InsCer), Porto Alegre, Rio Grande do Sul, Brazil; ^3^ Global Brain Health Institute (GBHI), San Francisco, CA, USA; ^4^ Neurology Department, São Lucas Hospital of PUCRS, Porto Alegre, Rio Grande do Sul, Brazil

## Abstract

**Background:**

Despite significant advances in diagnosis and the development of disease‐modifying therapies for dementia, low‐ and middle‐income countries (LMICs) face several challenges in these processes. In Brazil, the public health system — Unified Health System (SUS) — provides access to pharmacological treatments, including cholinesterase inhibitors, memantine, and psychotropic drugs. This study aims to analyze the prescription patterns of cholinesterase inhibitors, memantine and psychotropic medications, as well as their combinations, in an outpatient memory clinic.

**Method:**

We retrospectively analyzed 280 patients diagnosed with dementia who had at least one visit over the past 10 years at a public tertiary neurology memory clinic. Patients were classified into 14 groups based on their prescribed pharmacological treatment. Baseline demographic characteristics, including sex, age, years of education, and Mini‐Mental State Examination (MMSE) scores, were assessed.

**Result:**

The study population had a mean age of 74 years and an average education level of 3.1 years (Table 1). The most prescribed medication class was selective serotonin reuptake inhibitors (SSRIs), used by 55.1% of participants, followed by atypical antipsychotics (AAPs) (48.2%), cholinesterase inhibitors (45.4%), and memantine (22.9%) (Figure 1). Regarding therapeutic regimens, the most frequently employed was SSRI monotherapy (14.6%), followed by the combination of cholinesterase inhibitors, SSRIs, and atypical antipsychotics (10%). The least utilized prescriptions were memantine monotherapy (2.1%) and memantine combined with atypical antipsychotics (2.1%). Overall, 86.8% of patients accessed at least one specific drug for their disease or symptoms (Figure 2).

**Conclusion:**

Our findings showed that 86.6% of patients accessed at least one drug. However, less than 50% of them utilized dementia‐specific medications, which may be due to their indication being primarily for Alzheimer's disease or challenges in accessing these therapies within the Brazilian public health system. With the aim of enhancing therapeutic strategies and addressing existing gaps, further analyses will help elucidate the characteristics of these patients, their prescription patterns, and the barriers to improving dementia care.